# Variation in Cell Surface Hydrophobicity among Cryptococcus neoformans Strains Influences Interactions with Amoebas

**DOI:** 10.1128/mSphere.00310-20

**Published:** 2020-04-29

**Authors:** Raghav Vij, Carina Danchik, Conor Crawford, Quigly Dragotakes, Arturo Casadevall

**Affiliations:** a Department of Molecular Microbiology and Immunology, Johns Hopkins Bloomberg School of Public Health, Baltimore, Maryland, USA; b Centre for Synthesis and Chemical Biology, University College Dublin, Belfield, Ireland; Duke University Medical Center

**Keywords:** cell surface hydrophobicity (CSH), *Cryptococcus neoformans*, *Cryptococcus gattii*, *Acanthamoeba castellanii*, capsular antibody, polysaccharide capsule

## Abstract

Cryptococcus neoformans and Cryptococcus gattii are pathogenic fungi that cause significant morbidity and mortality. Cell surface hydrophobicity (CSH) is a biophysical parameter that influences the adhesion of fungal cells or spores to biotic and abiotic surfaces. C. neoformans is encased by polysaccharide capsule that is highly hydrophilic and is a critical determinant of virulence. In this study, we report large differences in the CSH of some C. neoformans and C. gattii strains. The capsular polysaccharides of C. neoformans strains differ in repeating motifs and therefore vary in the number of hydroxyl groups, which, along with higher-order structure of the capsule, may contribute to the variation in hydrophobicity that we observed. We found that cell wall composition, in the context of chitin-chitosan content, does not influence CSH. For C. neoformans, CSH correlated with phagocytosis by natural soil predator Acanthamoeba castellanii. Furthermore, capsular binding of the protective antibody (18B7), but not the nonprotective antibody (13F1), altered the CSH of C. neoformans strains. Variability in CSH could be an important characteristic in comparing the biological properties of cryptococcal strains.

**IMPORTANCE** The interaction of a microbial cell with its environment is influenced by the biophysical properties of a cell. The affinity of the cell surface for water, defined by the cell surface hydrophobicity (CSH), is a biophysical parameter that varies among different strains of Cryptococcus neoformans. The CSH influences the phagocytosis of the yeast by its natural predator in the soil, the amoeba. Studying variation in biophysical properties like CSH gives us insight into the dynamic host-predator interaction and host-pathogen interaction in a damage-response framework.

## Abstract (precis)

The interaction of a microbial cell with its environment is influenced by the biophysical properties of a cell. The affinity of the cell surface for water, defined by the cell surface hydrophobicity (CSH), is a biophysical parameter that varies among different strains of Cryptococcus neoformans. The CSH influences the phagocytosis of the yeast by its natural predator in the soil, the amoeba. Studying variation in biophysical properties like CSH gives us insight into the dynamic host-predator interaction and host-pathogen interaction in a damage-response framework.

## INTRODUCTION

The encapsulated Basidiomycetes that comprise the *Cryptococcus* species complex include several pathogenic species including Cryptococcus neoformans and Cryptococcus gattii. *Cryptococcus* spp. have a worldwide geographic distribution and are unusual among fungal pathogens in that they have polysaccharide capsules that are essential for mammalian virulence.

Human infection usually begins in the lung. Infectious propagules of C. neoformans, in the form of spore or yeast, may be inhaled to cause a pulmonary infection that is usually cleared in immunocompetent hosts or becomes latent. Conditions that impair immunity, such as HIV infection, are associated with disseminated disease, which usually manifests clinically as a meningoencephalitis. Recent evidence suggests that the nature of the infectious propagule has a significant effect on the outcome of the infection, as spores from C. neoformans cause significantly higher fungal burden in the brain of a murine model than small encapsulated yeast (1).

C. neoformans has been isolated from avian guano, soil, or arboreal sources. C. gattii has been isolated from trees, soil, freshwater, and seawater. There are three serotypes of C. neoformans, now referred to as Cryptococcus neoformans var. *neoformans* (serotype D), Cryptococcus neoformans var. *grubii* (serotype A), and hybrid (serotype AD). Phylogenetic evidence suggests that they may be classified as separate species, C. neoformans, Cryptococcus deneoformans, and hybrid, respectively (2). Interestingly, C. neoformans var. *grubii* has been isolated from 63% of clinical samples collected worldwide, followed by C. neoformans hybrid (6%) and C. neoformans var. *neoformans* (5%) (3, 4). The genomic diversity in the cryptococcal species complex may contribute to differences in the biophysical properties of cell surfaces within the *Cryptococcus* species complex.

C. neoformans and C. gattii cells are surrounded by a polysaccharide capsule that can dramatically vary in size during infection (5) and helps the pathogen evade the mammalian immune system. Highly branched polysaccharides (6) radiate outward from the cell wall, to form a dense matrix whose porosity increases with the distance from the cell wall, with reducing ends localized at the cell wall (7, 8). The capsule is primarily composed of glucuronoxylomannan (GXM; 98%), along with minor components glucuronoxylomannogalactan (GXMGal) and mannoproteins. GXM contains a core repeating structure of a α-(1→3)-mannose triad, with a β-(1→2) glucuronic acid branch on every third mannose (9). The capsules of different serotypes of C. neoformans and C. gattii have distinguishable polysaccharide motifs characterized by a varied degree of β-(1→2) or β-(1→4) xylose substitutions, and 6-*O*-acetyl substitutions along the mannan backbone (10). Polysaccharides are highly enriched in hydroxyl groups and form an extensive network of intramolecular and intermolecular hydrogen bonds, which includes bonding with water molecules. Therefore, polysaccharides are intrinsically hydrophilic molecules, which could provide an explanation for approximately 95% of the capsule’s weight (11). Structure-function relationships in glycans are poorly understood, but branching and substitution of polysaccharides likely affect the intra- and intermolecular hydrogen bonds, and therefore the rigidity of the polymer, thereby affecting the polysaccharide’s ability to form hydrogen bonds with water, which would result in variation of the conformation and hydrophobicity (12–15).

Factors that affect the biophysical parameters of the microbial surface of the *Cryptococcus* species complex have been previously described. For instance, melanization, capsule induction, and binding of capsular antibody alter the cell surface charge, which also varies by strain (16). Chronological aging of the yeast and antibody binding alter the elasticity of the polysaccharide capsule that surrounds the C. neoformans cell (17, 18).

**FIG 1:**
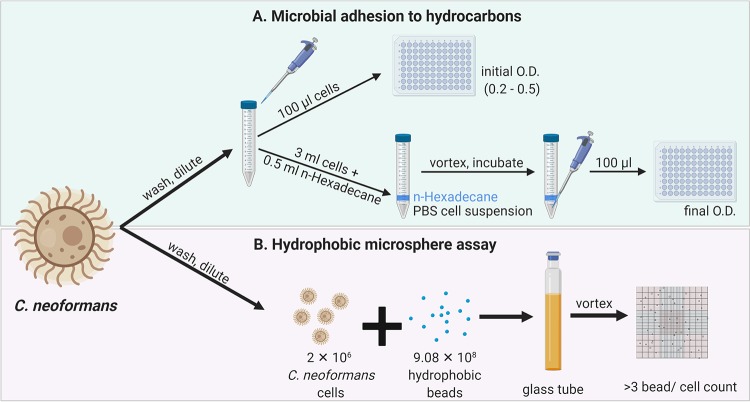
Methods for estimation of C. neoformans CSH. (A) CSH estimated by MATH assay that quantifies the interaction of C. neoformans cells in a suspension with the hydrocarbon solvent *n*-hexadecane. CSH% was calculated as the percent change in OD of a C. neoformans cell suspension after vortexing the mixture of cells with *n*-hexadecane. (B) In addition, we estimated CSH by visualizing the interaction between C. neoformans cells and hydrophobic beads (0.8 μm) in a hemocytometer and counting cells that had >3 beads/100 cells to calculate CSH%. Image created with BioRender.

Cell surface hydrophobicity (CSH) is a property of a microbial surface that reflects the affinity of components of the microbe’s cell surface for water and is calculated by estimating the affinity of cell surfaces for hydrophobic substances like hydrophobic columns, solvents, or polystyrene beads (Fig. 1). The biological role of CSH has been studied in bacteria such as Staphylococcus aureus and some fungi and has been succinctly reviewed (19). Previous studies of Candida albicans have established the importance of CSH for the interaction of the pathogen with the host tissue (20). Furthermore, strain-specific variation in CSH of clinical isolates and variation between species of *Candida* species complex have been reported (21).

The biophysical properties of the infectious propagule of C. neoformans in the form of yeast or spore influence the interaction of the yeast with its environment and inside the host during infection. For example, during infection, C. neoformans interacts with lung epithelial cells and macrophages and can pass through the blood-brain barrier. In the environment, *Cryptococcus* species complex is believed to interact with amoebas (22) and nematodes (23). Furthermore, hydrophobicity may influence the phagocytosis of microbial cells or particles by amoebas (24).

In this study, we report variation in CSH of C. neoformans and C. gattii strains using two independent methods. Further, we observed that CSH correlated positively with phagocytosis by Acanthamoeba castellanii. Additionally, the higher-order structure of the capsule is affected by the different capsular polysaccharide motifs that vary between serotypes of C. neoformans and C. gattii, which may influence the CSH. We also found that binding of protective but not nonprotective antibodies altered the hydrophobicity of C. neoformans grown in capsule induction medium.

## RESULTS

### Cryptococcal species manifest significant differences in CSH.

**FIG 2:**
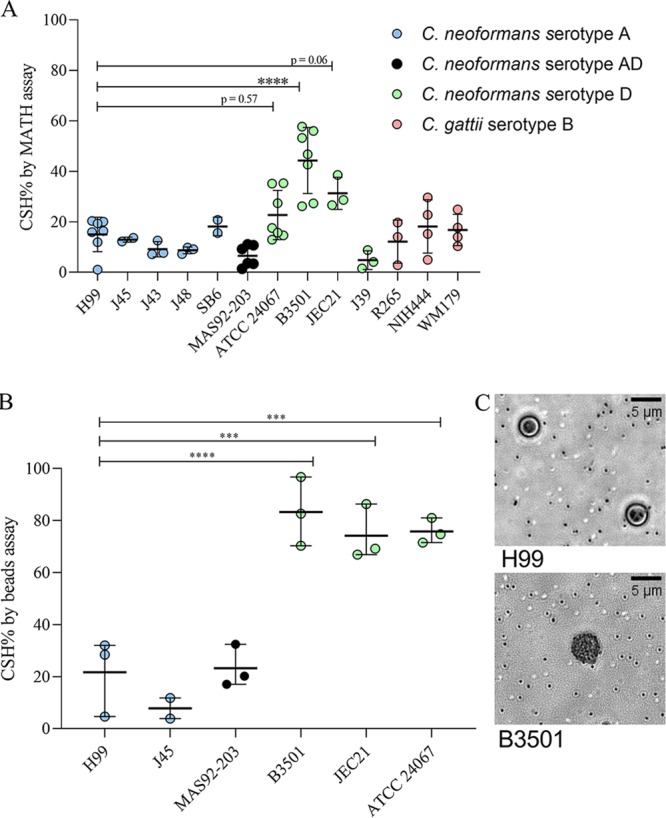
CSH of C. neoformans differs by strain. Graphical representation of CSH of C. neoformans and C. gattii strains. (A) Graphical representation of CSH estimated by MATH assay. (B) Graphical representation of CSH estimated by hydrophobic microsphere assay (left). Experiments have been performed 2 to 6 times independently, as indicated by individual data points. Circles indicate data points of CSH of an encapsulated strain of C. neoformans and C. gattii. Error bars represent the standard deviation of the mean. (C) Representative image of a mixture of hydrophobic beads with C. neoformans strain H99 (top) and relatively hydrophobic C. neoformans strain B3501 (bottom) used for the assay. Hydrophobic beads (small spheres, approximately 0.8 μm in diameter) adhere to the cell surface due to the high hydrophobicity of the B3501 cell, covering it almost completely. The hydrophobic beads are all but absent from the surface of H99 cells. Ordinary one-way analysis of variance was used to compare the CSH of C. neoformans strain H99 with the CSH of C. neoformans and C. gattii strains. The following symbols were used to annotate the statistical significance of the results: ***, *P* ≤ 0.001; ****, *P* ≤ 0.0001.

Measuring CSH by the MATH (microbial adhesion to hydrocarbons) and hydrophobic microsphere techniques (Fig. 1) revealed considerable variability among cells of C. neoformans and C. gattii strains cultured in Sabouraud dextrose broth (Fig. 2). By MATH assay, we found that serotype D strains B3501 and JEC21 were significantly more hydrophobic than the reference strain H99 (Fig. 2A). By the hydrophobic microsphere assay, we found that all strains of serotype D for which CSH was estimated, including B3501, ATCC 24067, and JEC21, were significantly more hydrophobic than the reference strain H99 (Fig. 2B). However, there was considerable strain-to-strain variation, and no pattern emerged regarding differences between serotypes or species, except for the notable finding that the most strains manifesting highest CSH were C. neoformans serotype D.

### Capsule composition may influence the CSH.

**FIG 3:**
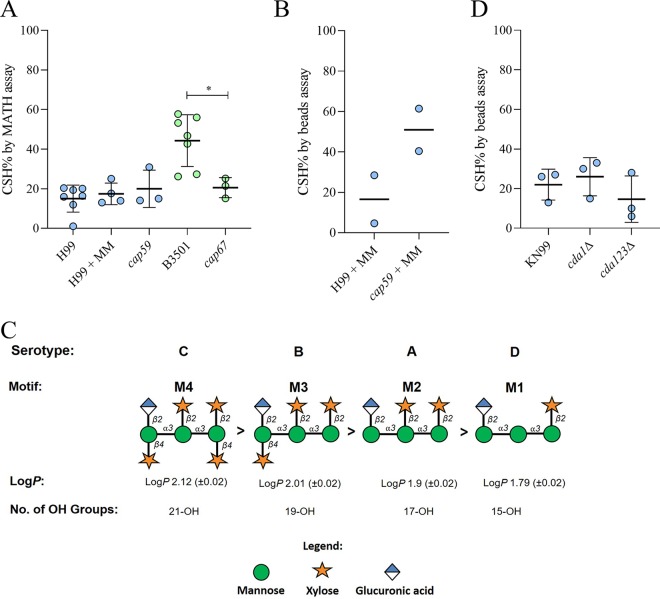
Capsular motifs, and not the cell wall composition, may contribute to the variation in hydrophobicity of C. neoformans. (A) Comparison of the CSH of C. neoformans H99 grown in Sabouraud broth with those of C. neoformans H99 grown in MM and acapsular *cap59* strain and comparison of the CSH of strain B3501 with that of acapsular *cap67* strain. One-way analysis of variance and *t* test were used, respectively, to compare the means. Data have been compiled from separate experiments to draw comparisons and study the influence of presence or induction of capsule on the CSH of C. neoformans. Each data point represents a biological replicate, and the error bar represents the SD of the mean. (B) CSH% measured by a hydrophobic bead assay of C. neoformans strain H99 and acapsular strain *cap59* grown in capsule induction medium. The experiment was performed in two independent replicates, as represented by data points about the median. (C) Lipophilicity, log *P*, of dominant carbohydrate motifs in the carbohydrate was predicted by an equation proposed by Mannhold et al. (28). M4 was found to be the most hydrophobic motif and M1 the least. The number of hydroxyl groups on each polysaccharide motif was calculated (below). Glycan notification followed the Symbol Nomenclature for Glycans (SNFG) (71). (D) No significant differences were found when the CSH% of C. neoformans strain K99 was compared to those of chitin deacetylase 1 mutant (*cda1Δ*) and chitin deacetylase triple-knockout mutant (*cda123Δ*) strains as measured by hydrophobic microsphere assay. The following symbols were used to annotate the statistical significance of the results: *, *P* ≤ 0.05.

The capsule that surrounds the C. neoformans cell is thought to be highly hydrophilic and is primarily composed of water (11). Hence, we sought to ascertain its contribution to CSH in C. neoformans strain H99 (serotype A) by comparing encapsulated H99 and the nonencapsulated *cap59* strain. To our surprise, we observed no major difference in CSH between H99 and *cap59* cells grown in Sabouraud dextrose broth, by the MATH assay (Fig. 3A). However, when grown in capsule-inducing minimum medium (25), the nonencapsulated strain bound more hydrophobic beads than the encapsulated strains (Fig. 3B). Next, we compared the CSH of C. neoformans strain B3501 (serotype D) to the acapsular *cap67* mutant, on the B3501 background (26, 27). We observed a significant decrease in the CSH by MATH assay (Fig. 3A).

Different strains and serotypes of C. neoformans and C. gattii have different dominant carbohydrate motifs in their capsule (10) that may influence the experimentally observed variation in CSH. To test this hypothesis, we used *in silico* method described by Mannhold et al. (28), to calculate and compare the lipophilicity (log *P*) of the four dominant GXM motifs. We observed the following trend in the predicted lipophilicity of GXM carbohydrate motifs: M4 (dominant in serotype C, log *P* 2.12) > M3 (dominant in serotype B, log *P* 2.01) > M2 (dominant in serotype A, log *P* 1.9) > M1 (dominant in serotype D, log *P* 1.79) (Fig. 3C).

Based on the rationale that polysaccharides enriched in a greater number of hydroxyl groups would have higher hydrophilicity, we counted the number of hydroxyl groups of each dominant GXM motif (Fig. 3C). The M4 motif (dominant in serotype C) contained the highest number of hydroxyl groups, 21, followed by 19 hydroxyl groups in M3 (dominant in serotype B), 17 hydroxyl groups in M2 (dominant in serotype A), and 15 hydroxyl groups in M1 (dominant in serotype D).

Next, we wanted to investigate if the cell wall composition influences the CSH of C. neoformans. The enzymes chitin deacetylases, encoded by the *cda* gene family, deacetylate chitin, a hydrophobic polymer that is an important cell wall component, to chitosan, a hydrophilic polymer (29). We found no significant differences between wild type and strains knocked out for *cda1* and for *cda1*, *cda2*, and *cda3* (Fig. 3D).

### CSH of unopsonized C. neoformans correlates with phagocytosis by A. castellanii.

**FIG 4:**
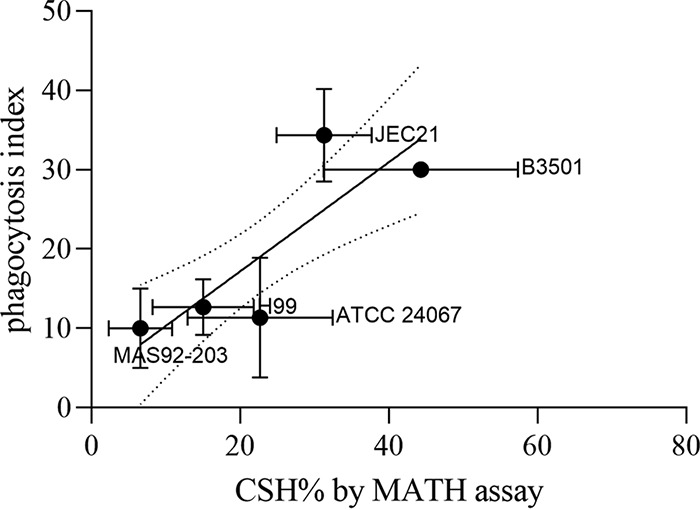
CSH of C. neoformans correlates with phagocytosis of C. neoformans by natural predator A. castellanii. Significant positive linear correlation (*R*^2^ = 0.5722) between CSH of C. neoformans strains and phagocytosis index by A. castellanii. Phagocytosis index is estimated by fluorescence microscopy as the number of C. neoformans cells labeled by Uvitex internalized per 100 A. castellanii cells. Error bars represent the standard deviation of the mean.

To test whether CSH influences phagocytosis by soil predators like the amoeba, we incubated fungal and protozoal cells and estimated the phagocytosis index. We found a positive and linear correlation between CSH of C. neoformans strains and phagocytosis index of C. neoformans strains by A. castellanii (Fig. 4). In this series of isolates, the phagocytosis index also correlated with strain serotype.

### Effect of antibody binding on CSH.

**FIG 5:**
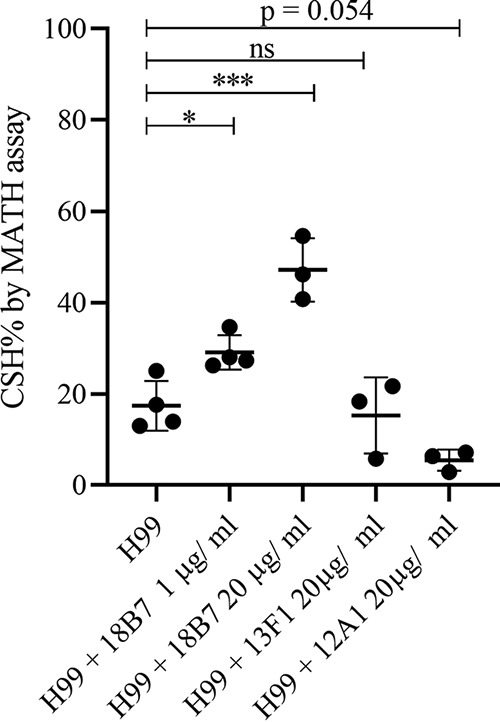
Binding of protective capsule antibodies influences CSH. Incubation of C. neoformans strain H99 grown in the capsule induction medium (MM) with protective capsular antibody 18B7 significantly increased CSH in a concentration-dependent manner, while 12A1 decreased CSH and 13F1 had no significant effect on CSH. CSH was determined by MATH assay in 2 to 3 biological replicates, as indicated by data points. Error bars represent the standard deviation of the mean. Ordinary one-way analysis of variance was used to compare the CSH of untreated C. neoformans strain H99 with the CSH of H99 cells treated with different antibodies. The following symbols were used to annotate the statistical significance of the results: ns, *P* > 0.05; *, *P* ≤ 0.05; ***, *P* ≤ 0.001.

Previous studies have demonstrated that capsule antibody binding alters capsule structure and changes the surface charge of C. neoformans (16, 17). This led us to investigate the effect of binding of capsular antibodies to C. neoformans on the CSH. We demonstrated that binding of capsular antibody 18B7 (30) increases CSH in a concentration-dependent manner, while binding of nonprotective antibody 13F1 has no significant effect on the CSH of C. neoformans cells grown in the capsule induction medium (Fig. 5).

## DISCUSSION

In this study, we measured the CSH of C. neoformans and found considerable interstrain variation. When CSH was estimated by hydrophobic microsphere assay, C. neoformans serotype D strains were likely to be more hydrophobic than C. neoformans serotype A strains, with the caveat that we analyzed a relatively small set of strains from each serotype. We also demonstrated that CSH is a biophysical parameter that may influence the interaction of yeast cells with the environmental predator Acanthamoeba castellanii. Finally, we demonstrated that the binding of a protective capsular antibody alters the CSH.

An earlier study suggested that capsule and protective antiserum binding influenced hydrophobicity of C. neoformans (31). They reported no correlation between CSH and phagocytosis of C. neoformans by mouse peritoneal macrophages (31). The difference between our observations and the prior report may be attributed to the differences in methodologies. In the prior study, hydrophobicity was estimated from the number of cells that bound to hydrophobic columns, and the cells were fixed with formalin, which may have altered surface properties of the yeast. In this study, we have used the MATH assay, which relies on the interaction of microbes with hydrophobic solvents to calculate CSH (Fig. 1A) (32). In addition, we have used a hydrophobic microsphere assay that quantitates the interaction between hydrophobic beads and the yeast, visualized under a bright-field microscope, to estimate the CSH (Fig. 1B) (20).

C. neoformans polysaccharides, like GXM, are essential components required in the formation of microbial communities called biofilms that are protective for the fungi (33). C. neoformans biofilms have been reported on medical devices (34, 35). Biofilm-associated cells have been associated with increased tolerance against antifungal drugs and phagocytic cells, as they upregulate proteins associated with host defense (36–38). *In vivo*, C. neoformans forms biofilm-like structures called cryptococcomas that could play a role in its neurotropism (39). The surface property of cells may affect the aggregation of microbial communities in biofilms. Interestingly, ATCC 24067 and B3501 strains, which are highly hydrophobic, also form biofilms more easily than the H99 strain, which is relatively less hydrophobic (Fig. 1) (36, 37). A similar correlation between the formation of biofilm and CSH was observed in *Candida* spp. (21, 40, 41) and in bacteria (42). Flocculation, another multicellular phenotype observed in yeasts, has been observed in C. neoformans cells during growth in certain media (43) and could be caused by changes in CSH, as reported for brewer’s yeast (44).

Amoebas are natural predators of *Cryptococcus* species (22, 45) and have emerged as a powerful tool for studying mechanisms of intracellular pathogenesis and evolution of virulence (46, 47). A growing body of evidence suggests that virulence traits have emerged in environmental fungi, including *Cryptococcus* species, because of the selection pressure that results from fungus-amoeba interaction (48). Our finding that the more hydrophobic *Cryptococcus* strains were more readily phagocytosed is congruent with the observation that amoebas can phagocytose hydrophobic particles (24), although these mechanisms are not well understood. There is a remarkable correspondence between C. neoformans virulence traits that influence phagocytosis and enable survival of the fungi in A. castellanii and in human macrophages (46). For instance, the capsule of C. neoformans masks cell wall components that are recognized by innate immune receptors (49), and the absence of capsule leads to poor survival of C. neoformans incubated with A. castellanii (46). *In vitro* studies of macrophage and C. neoformans interaction usually require opsonins such as capsular antibodies and complement (50, 51) for phagocytosis by innate immune cells. Since opsonins change the CSH of C. neoformans and without opsonins phagocytosis of cryptococcal cells by macrophages is essentially nil (52), comparable studies with mammalian phagocytic cells are difficult to do. Studying the effect of CSH on phagocytosis in amoebas may give insights into factors independent of opsonin-receptor interaction that may influence phagocytosis in macrophages.

Murine antibodies that recognize capsular epitopes of C. neoformans can confer passive protection on the host and enhance macrophage activity (53, 54). In addition to facilitating phagocytosis of the yeast, the murine IgG antibody 18B7 (30) alters capsule stiffness and impairs cellular replication of the yeast (17), significantly alters the cell surface charge (16), and has a catalytic activity that breaks down the capsule (55). In this study, we report that monoclonal antibody (MAb) 18B7 binding significantly increased the hydrophobicity of the cryptococcal cell surface in a concentration-dependent manner, while a nonprotective antibody, IgM 13F1, did not alter the CSH. We may attribute the differential effect of changes in CSH induced by MAbs 13F1 and 18B7 to the pattern of MAb binding, since MAb 18B7 binds near the surface in an annular pattern (17, 30, 56), while MAb 13F1 binds throughout the capsule in a punctate pattern (57, 58). There is precedence for our observation in the encapsulated bacterium Klebsiella aerogenes, where the pattern of diffusion of some MAbs through the polysaccharide capsule has been shown to influence the cell surface hydrophobicity (59, 60).

A surprising result in our study was that some C. neoformans strains manifest a considerably higher CSH than others, despite being surrounded by a hydrophilic capsule. The origin and mechanism for variability in CSH in these strains are not understood. Glycans are intrinsically hydrophilic molecules. Lipophilicity for glycans may be described by the partition coefficient (*P*), which is quantified as the distribution of a compound between two immiscible solvents, like water and octanol (61). While prior studies have compared the lipophilicities for monosaccharides, these efforts are not standardized in the field (14). For small molecules, log *P* can be accurately predicted by an equation proposed by Mannhold et al., although the accuracy of the prediction decreases with an increase in nonhydrogen atoms (28). In this study, we used this calculation to predict and compare the lipophilicities of capsular carbohydrate motifs (28), with the caveat that the suitability of these equations for molecules larger than monosaccharides is uncertain. The predicted calculated lipophilicity of GXM oligosaccharide motifs was positive, incorrectly suggesting that the polymers would preferentially partition into an organic solvent. The M1 motif, which is dominant on the C. neoformans serotype D strains, was found to be less lipophilic than M3 and M4 motifs that are dominant in C. gattii serotype B and C. neoformans serotype A strains, respectively (Fig. 3D). This goes against our experimental observation that some C. neoformans serotype D strains were more hydrophobic than serotype B and A strains (Fig. 2) and implies that simple calculations of lipophilicity do not explain our findings. Instead, we suspect that the measured hydrophobicity stems from higher-order polysaccharide structures that could present different molecular surfaces in their interaction with the solvent.

The dynamic and noncrystalline nature of polysaccharides makes it challenging to obtain defined structures and to relate the structure of glycans to their activity and biological roles. Yet, we know that the flexibility of the oligosaccharide polymer is influenced by intra- and intermolecular hydrogen bonds. Theoretical predictions suggest that α-(1→3)-mannan forms weak intermolecular hydrogen bonds, resulting in a polymer with a flexible structure that allows for many hydroxyl groups to interact with water (12). The primary component in the capsule of C. neoformans is built upon repeating α-(1→3)-mannose triads, which would contribute to the observation that 95% of capsule’s weight comes from water (11). We also found that the number of hydroxyl groups in each motif (Fig. 3C) was inversely related to the observed CSH. The dominant motif M1 in the capsule of C. neoformans serotype D had fewer hydroxyl groups and the strains of serotype D tend to have higher CSH, compared to the number of hydroxyl groups in dominant motifs M2 and M3 of serotype A and B, whose strains had comparatively lower CSH. Fewer hydroxyl groups result in fewer opportunities for hydrogen bonding between the polysaccharide and water, which could translate into fewer hydrophilic structures with higher CSH.

It is also important to note that the motifs that enrich the capsule may differ between strains of the same serotype (10). For example, in C. neoformans serotype D strain 24067, capsular polysaccharide chemotyping suggests that the M1 motif dominates 100% of the strain, while C. neoformans serotype D strain B3502 is composed of the dominant M1 (52%) motif and the M6 (48%) motif (10). This may contribute to the variation of CSH within strains grouped in serotype D (Fig. 2).

In addition, we sought to explore whether the composition of the cell wall, in particular the chitin-chitosan content in the cell wall, which is regulated by *cda* genes encoding chitin deacetylases (62), may influence the adhesion of C. neoformans to various surfaces (63). The role of these enzymes in adhesion has also been described for the plant-pathogenic fungus Magnaporthe oryzae (64). In C. neoformans, chitosan is reported to be important for cell wall integrity, such that mutants that lack the *cda* genes display attenuated virulence (29, 62, 65). In addition, while chitin is hydrophobic, the deacetylated form chitosan is relatively hydrophilic because it is enriched in amines, a hydrophilic functional group (66, 67). When we compared the hydrophobicity of mutants that are deficient in chitosan with that of the wild type, we found no significant differences. We also sought to alter the cell wall composition by inducing the formation of the hydrophobic pigment melanin in C. neoformans strain H99 by growing the cells with and without the catecholamine precursor l-3,4-dihydroxyphenylalanine (l-DOPA). We did not observe any notable difference between binding levels of hydrophobic beads to melanized and nonmelanized cells (experiment performed once; data not shown). Therefore, our data indicate that the capsular polysaccharide that surrounds the cell, and not the cell wall composition, influences the hydrophobicity of the C. neoformans cell surface. Lipophilic structures have been reported in the capsule, which might extend to the surface and influence the hydrophobicity of the cell surface (12, 68).

In summary, we report that CSH of *Cryptococcus* species can differ significantly depending on the strain. We have also demonstrated the correlation of the biophysical parameter CSH with the phagocytosis by A. castellanii and shown that protective antibodies that bind to the capsule of C. neoformans can influence the hydrophobicity of C. neoformans. The findings that C. neoformans strains differ in CSH and that changes to this cell surface property correlates with biological properties suggest that the investigation of how this parameter is established and maintained could provide new insights into capsular structure.

## MATERIALS AND METHODS

### Strains and culture of C. neoformans and C. gattii.

Cryptococcus neoformans and C. gattii strains (Table 1) stored as frozen stocks at −80°C were streaked onto Sabouraud agar plates and incubated at 30°C for 48 h. The plates were stored at 4°C for use up to 1 week. Multiple colonies were selected and inoculated into 5 ml of liquid medium and Sabouraud broth and incubated at 30°C with shaking for 2 days. For capsule induction, 10^6^ cells/ml were washed twice in phosphate-buffered saline (PBS) and inoculated into MM (10 mM MgSO_4_, 29.3 mM KH_2_PO_4_, 13 mM glycine, 3 μM thiamine-HCl, and 15 mM dextrose, with pH adjusted to 5.5).

**TABLE 1:** Strains of C. neoformans and C. gattii used in the present study

Species and strain	Mutant	Serotype	Source or reference^*a*^
Cryptococcus neoformans			
H99		A	John Perfect (Durham, NC)
	*cap59*		
K99			29
	*cda1*Δ		29
	*cda1,2,3*Δ		29
J45			72
J10			72
J43			72
J48			72
SB6			73
MAS92-203		AD	6
ATCC 24067		D	6
B3501			6
	*cap67*		27
JEC21			74
J39			72

Cryptococcus gattii			
R265		B	ATCC (Manassas, VA) (75)
NIH444			6
WM179			ATCC (Manassas, VA) (75)

a The references indicate the study in which the strains were serotyped or the study in which the strains used had been characterized by serotype.

### Antibody incubation.

C. neoformans (H99) grown in MM was washed twice in PBS. Protective and nonprotective capsular antibodies, 18B7, 12A1, and 13F1 (69), respectively, were incubated for 1 h at 30°C with shaking. The CSH percentage (CSH%) was determined by MATH and microsphere assays as detailed below.

### Estimation of CSH by MATH.

CSH was estimated by the MATH assay as described in reference 32. Yeast cultures were washed twice in PBS and resuspended in 3 ml of PBS at an estimated initial optical density (OD) of 0.2 to 0.4 recorded as *A*_0_. An 0.4-ml amount of *n*-hexadecane was added, and the mixture was vortexed for 30 s and incubated at 30°C for 2 min to allow the layers to separate. Final OD (*A*_1_) of the aqueous layer was recorded and estimated as an average from 3 technical replicates in a 96-well plate read by an Emax Plus microplate reader (Molecular Devices). CSH% was estimated as [1 − (*A*_0_/*A*_1_)] × 100.

### Estimation of CSH by hydrophobic microsphere assay.

CSHs of C. neoformans and C. gattii were estimated by the method detailed in reference 20 by adding 9.02 × 10^8^ 0.8-μm green hydrophobic beads (Bang Laboratories) to 2 ml of 2 × 10^6^ cells/ml in sodium phosphate buffer (0.05 M, pH 7.2) in clean glass tubes. After equilibration at room temperature (RT) for 2 min, the mixture was vortexed vigorously for 30 s. Cells were imaged at 40×, 100 cells were counted, and the percentage of cells having >3 attached microspheres was considered the CSH% value.

### Acanthamoeba castellanii culture.

Acanthamoeba castellanii strain 30234 was obtained from the American Type Culture Collection (ATCC). Cultures were maintained in PYG broth (ATCC medium 712) at 25°C according to instructions from ATCC.

### Acanthamoeba castellanii phagocytosis index.

The phagocytosis index was estimated as detailed in reference 70 with minor modifications. Briefly, 5 × 10^5^ cells/ml of A. castellanii were incubated in 35-mm no. 1.5 coverslip MatTek dishes with Dulbecco’s PBS (DPBS) (Ca^2+^ and Mg^2+^) for 3 to 4 h. C. neoformans or C. gattii strains were incubated with 10 μg/ml Uvitex (fungal cell wall dye), inoculated at a multiplicity of infection (MOI) of 1 and incubated for 2 h at 25°C. The cells were imaged using a Zeiss Axiovert 200M inverted microscope with a 20× phase objective. Phagocytosis index was estimated by counting the number of C. neoformans or C. gattii cells engulfed per 100 amoeboid cells.

### Estimation of lipophilicity and number of hydroxyl groups in carbohydrate motifs.

The lipophilicity of the carbohydrate motif dominant in the capsule of an C. neoformans serotype was estimated by the method described by Mannhold et al. (28), as the log of the partition coefficient (*P*): log *P* = 1.46 (±0.02) + 0.11 (±0.001)NC − 0.11 (±0.001)NHET, where NC is the number of carbon atoms in a molecule and NHET is the number of hetero atoms.

The number of hydroxyl groups in each motif of C. neoformans capsule was counted manually, as proxy for the number of hydrogen bond donor and acceptor atoms.
